# Spatiotemporal progression of ubiquitin-proteasome system inhibition after status epilepticus suggests protective adaptation against hippocampal injury

**DOI:** 10.1186/s13024-017-0163-2

**Published:** 2017-02-24

**Authors:** Tobias Engel, Jaime Martinez-Villarreal, Christine Henke, Eva M. Jimenez-Mateos, Amaya Sanz-Rodriguez, Mariana Alves, Yasmina Hernandez-Santana, Gary P. Brennan, Aidan Kenny, Aoife Campbell, Jose J. Lucas, David C. Henshall

**Affiliations:** 1 Department of Physiology & Medical Physics, Royal College of Surgeons in Ireland, 111 St. Stephen’s Green, Dublin, 02 YN77 Ireland; 2 Medical Clinic III, University Clinic Dresden, TU Dresden, Dresden, Germany; 3 Centro de Biología Molecular “Severo Ochoa”, Consejo Superior de Investigaciones Científicas, Universidad Autónoma de Madrid, Madrid, Spain; 4 Networking Research Center on Neurodegenerative Diseases (CIBERNED), Instituto de Salud Carlos III, Madrid, Spain

**Keywords:** Ubiquitin-proteasome system, *Status epilepticus*, Epilepsy, Proteasome inhibition, Neuroprotection, Hippocampal sclerosis, Epileptogenesis

## Abstract

**Background:**

The ubiquitin-proteasome-system (UPS) is the major intracellular pathway leading to the degradation of unwanted and/or misfolded soluble proteins. This includes proteins regulating cellular survival, synaptic plasticity and neurotransmitter signaling; processes controlling excitability thresholds that are altered by epileptogenic insults. Dysfunction of the UPS has been reported to occur in a brain region- and cell-specific manner and contribute to disease progression in acute and chronic brain diseases. Prolonged seizures, *status epilepticus*, may alter UPS function but there has been no systematic attempt to map when and where this occurs in vivo or to determine the consequences of proteasome inhibition on seizure-induced brain injury.

**Method:**

To determine whether seizures lead to an impairment of the UPS, we used a mouse model of *status epilepticus* whereby seizures are triggered by an intra-amygdala injection of kainic acid. *Status epilepticus* in this model causes cell death in selected brain areas, in particular the ipsilateral CA3 subfield of the hippocampus, and the development of epilepsy after a short latent period. To monitor seizure-induced dysfunction of the UPS we used a UPS inhibition reporter mouse expressing the ubiquitin fusion degradation substrate ubiquitin^G76V^-green fluorescent protein. Treatment with the specific proteasome inhibitor epoxomicin was used to establish the impact of proteasome inhibition on seizure-induced pathology.

**Results and conclusions:**

Our studies show that *status epilepticus* induced by intra-amygdala kainic acid causes select spatio-temporal UPS inhibition which is most evident in damage-resistant regions of the hippocampus, including CA1 pyramidal and dentate granule neurons then appears later in astrocytes. In support of this exerting a beneficial effect, injection of mice with the proteasome inhibitor epoxomicin protected the normally vulnerable hippocampal CA3 subfield from seizure-induced neuronal death in the model.

These studies reveal brain region- and cell-specific UPS impairment occurs after seizures and suggest UPS inhibition can protect against seizure-induced brain damage. Identifying networks or pathways regulated through the proteasome after seizures may yield novel target genes for the treatment of seizure-induced cell death and possibly epilepsy.

**Electronic supplementary material:**

The online version of this article (doi:10.1186/s13024-017-0163-2) contains supplementary material, which is available to authorized users.

## Background

The cell and molecular mechanisms by which injury to the brain leads to the later emergence of chronic, spontaneous seizures, a process termed epileptogenesis, remains incompletely understood [1]. Epileptogenesis is associated with large-scale changes to gene expression which is thought to impact on neuronal survival and to drive remodelling of neuronal networks, aberrant neurotransmitter receptor expression and function, gliosis, neuroinflammation and other characteristic changes [2]. It is increasingly recognized that targeting single genes is unlikely to be sufficient to disrupt epileptogenesis and ways to target larger signalling networks are required [1]. This has driven a strong focus on transcription factors and epigenetic mediators [3], systems genetics approaches [4] and post-transcriptional gene silencing by non-coding microRNAs [5]. This is apt because it is mRNA transcription that provides the greatest contribution to the abundance of proteins during stress [6, 7]. In contrast, protein turnover has been reported to contribute less than 10% to overall protein abundance [7]. Nevertheless, protein aggregates and dysfunction of the molecular machinery responsible for regulating protein turnover is evident in numerous neurodegenerative and neurologic disorders [8–10]. Thus, protein turnover may be especially important for the pathogenesis of diseases associated with the brain. Whether altered protein turnover is important in epileptogenesis is unknown.

The ubiquitin proteasome system (UPS) is the major intracellular pathway leading to the degradation of aberrant and/or misfolded soluble proteins [11]. The UPS is a highly conserved and tightly controlled ATP-consuming proteolytic system. Ubiquitin (Ub) is first activated by an E1 enzyme, and then transferred to an ubiquitin-conjugating E2 enzyme [11]. Substrates to be degraded are selectively recognized by E3 Ub ligases [12]. E3 ligases mediate the subsequent transfer of activated Ubs from E2 to the substrate leading to an increasing polyubiquitination chain [11]. Once Ub moieties reach four or more per substrate chain, the substrate is usually recognized by the 26S proteasome [13]. The 26S proteasome is composed of two 19S regulatory protein complexes important for substrate recognition and deubiquitination and a barrel-like shaped 20S protein complex with a catalytic core in charge of substrate cleavage into smaller peptides and amino acids [14].

The UPS is present and functions in multiple sub-cellular compartments including cytoplasm, nucleus, mitochondria, cell membranes and pre- and post-synaptic compartments in neurons [15–17]. In the nervous system, the UPS has been shown to regulate turnover of proteins critical for neuronal survival and neurotransmission [16, 17] by controlling the presence of functional glutamate [18] and γ-amino butyric acid (GABA) receptor levels [19], spinogenesis [20], dendrite growth and arborisation [21] and the formation of new synapses [22]. Proteasome inhibition has been proposed to play a causative role in numerous acute and chronic diseases of the brain including ischemia [10] and neurodegenerative diseases such as Alzheimer’s, Huntington’s and Parkinson’s [9, 23]. Consequently, UPS-targeting drugs have been proposed as potential therapeutic treatment strategies for various brain diseases [9].

Emerging evidence suggests the function and dysregulation of the UPS is important in epilepsy. Glutamate-induced excitotoxicity leads to an inhibition of the proteasome [24]. Genes linked to ubiquitin metabolism are prominent among transcripts regulated by seizures in animal models [25]. The deubiquitinating enzyme USP9X regulates excitability and seizures in animals and humans [26]. Genetic or pharmacologic targeting of the E3 ubiquitin ligase CRL4A (CRBN) increases seizure susceptibility [27]. The immunoproteasome, a particular form of the proteasome believed to play a key role during inflammation, is upregulated in brain tissue from patients with temporal lobe epilepsy (TLE) [28] and in the hippocampus of rats with chronic epilepsy [29]. Lafora disease, a progressive myoclonic epilepsy characterized by the presence of glycogen-like intracellular inclusions named Lafora bodies, results from mutations in the ubiquitin E3 ligase malin [30]. In addition, 20S proteasome subunits have been shown to be sequestered in Lafora bodies [31]. Mutations in the E3 ligase Ube3a have been shown to cause Angelman syndrome, a rare neurological disorder commonly associated with intractable seizures [32] and the E3 ligase MDM2 controls levels of the transcription factor p53 which regulates cell death and seizure thresholds in experimental and human epilepsy [33, 34].

However, up to date there have been no studies carried out to characterize where exactly UPS inhibition occurs after seizures in vivo, which cell-types are involved and whether proteasome inhibition impacts on seizure-induced brain damage. Here we used a UPS inhibition green fluorescent protein (GFP)-reporter mouse [35] to map UPS inhibition in vivo following *status epilepticus*. We also tested a proteasome inhibitor for effects on seizure-induced neuronal death.

## Results

### Increase in polyubiquitinated conjugates in the hippocampus after *status epilepticus*

**Fig. 1:**
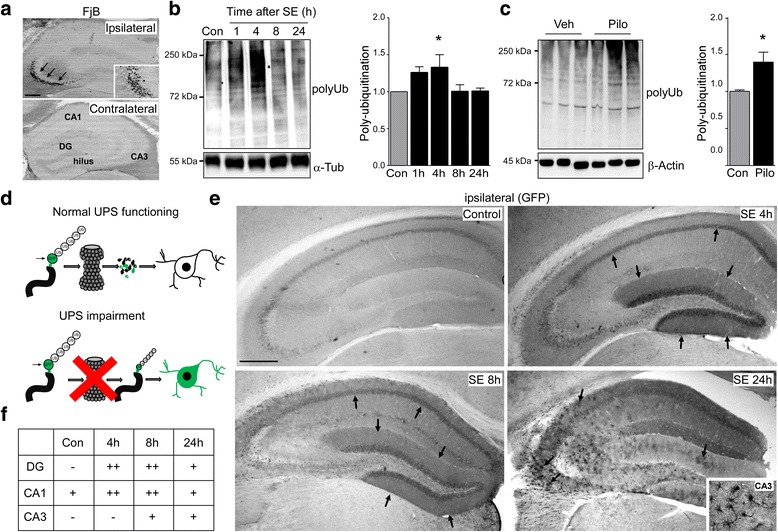
Increased polyubiquitination after *status epilepticus*. **a.** Representative pictures of coronal brain sections at the level of the dorsal ipsilateral and contralateral hippocampus 24 h after *status epilepticus* showing FjB-positive cells restricted mainly to the ipsilateral CA3 subfield (arrows and insert). Note, the ipsilateral CA1 and dentate gyrus (DG) and the contralateral hippocampus are mainly spared from cell death. Scale bar, 500 μm **b.** Representative Western (*n* = 1 per lane) and graph showing increased polyubiquitination levels (FK2 antibody) in the ipsilateral hippocampus after intra-amygdala KA induced-*status epilepticus* (mean ± sem, *p <0.05 by one-way ANOVA with Fisher’s post hoc test; *n* = 4 per group). **c**. Representative Western (*n* = 1 per lane) and graph showing increased polyubiquitination levels (FK2 antibody) in the hippocampus after pilocarpine-induced *status epilepticus* (mean ± sem, *p <0.05 by student’s two-tailed *t*-test; *n* = 4 per group). **d**. Schematic showing transgenic approach to monitor UPS impairment in vivo. Ub^G76V^-GFP transgenic mice over-express a modified green fluorescent protein (GFP) constitutively targeted for ubiquitin-dependent proteasome degradation. Under physiological conditions GFP is immediately degraded by the proteasome and no intracellular accumulation of GFP can be detected; however, if UPS function is compromised, GFP accumulates inside the cell where it can be detected. **e**. Representative photomicrographs (5x lens) showing subfield-specific increased GFP-reporter accumulation in the ipsilateral hippocampus of transgenic Ub^G76V^-GFP mice subjected to intra-amygdala KA induced *status epilepticus*. Note, strong GFP signal in the DG and CA1 hippocampal subfield shortly after *status epilepticus* (4 h and 8 h) and scattered GFP staining throughout the hippocampus involving all subfields 24 h after seizure suppression. Scale bar, 500 μm **f**. Table showing hippocampal (DG, CA1 and CA3) subfield-specific increase in GFP signal in transgenic Ub^G76V^-GFP mice after *status epilepticus*. Subfield-specific GFP signal was quantified according to a semi-quantitative scale after staining with GFP antibody (− baseline (absent/very low signal), + increase above baseline, ++ strong increase above baseline; *n* = 3 per group)

To determine whether prolonged seizures (*status epilepticus*) lead to an impairment of the UPS, we used our well characterized mouse model of *status epilepticus* [36]. In this model, *status epilepticus* is induced by a unilateral microinjection of kainic acid (KA) into the basolateral amygdala. Shortly after KA injection, mice develop continuous electrographic seizures (*status epilepticus)*. To reduce morbidity and mortality, mice are treated with the anticonvulsant lorazepam 40 min post-KA [36]. Increased transcript levels of the neuronal activity-regulated gene *c-Fos* in the ipsilateral hippocampus confirmed the recruitment of hippocampal structures during seizures (Additional file 1: Figure S1). In this model, seizure-induced cell death is mainly restricted to the ipsilateral hippocampus, in particular the CA3 subfield, as detected by Fluoro-Jade B (FjB) staining (Fig. 1a and [36]). The ipsilateral CA1 subfield and the granule neurons of the dentate gyrus (DG) are typically spared significant injury as are the subfields in the contralateral hippocampus (Fig. 1a and [36]).

To investigate whether the model is associated with an impairment of the UPS, we analyzed levels of polyubiquitinated conjugates in the ipsilateral hippocampus at different time-points after *status epilepticus*. Western blotting showed an early accumulation of polyubiquitinated substrates shortly after *status epilepticus* (Fig. 1b). To check this was not a model-specific characteristic, we performed an analysis of the hippocampus of mice subject to *status epilepticus* triggered by the muscarinic agonist pilocarpine [37]. As with the intra-amygdala KA model, pilocarpine-induced seizures resulted in accumulation of polyubiquitinated conjugates in the hippocampus (Fig. 1c).

### Subfield-specific UPS impairment in the hippocampus after *status epilepticus*

To explore when and where UPS impairment occurs in the hippocampus after *status epilepticus*, we made use of an UPS reporter mouse expressing modified GFP (Ub^G76V^-GFP). In this model, GFP is constitutively targeted for ubiquitin-dependent degradation by the proteasome through a ubiquitin fusion degradation (UFD) signal consisting of an N-terminal linked ubiquitin moiety accepting polyubiquitination chains thereby targeting GFP for its rapid degradation by the proteasome [38] (Fig. 1d). As reported previously, control Ub^G76V^-GFP mice showed a low number of GFP-positive cells detected by antibodies against GFP [39], with basal GFP staining most evident in the CA1 subfield of the hippocampus (Fig. 1e and f). Analysis of hippocampal subfield-specific *GFP* mRNA transcription and chymotrypsin-like proteasome activity under basal conditions showed that this was independent of differences in *GFP* transcription or intrinsic proteasome activity (Additional file 1: Figure S2A, B). In contrast, Ub^G76V^-GFP mice subjected to *status epilepticus* showed a strong increase in GFP immunoreactivity throughout the hippocampus (Fig. 1e). Unexpectedly, an increased GFP signal was first observed 4 and 8 h after *status epilepticus* in the ipsilateral DG and CA1 subfields, brain regions normally resistant to seizure-induced cell death in the model (Fig. 1e and f). In contrast, GFP levels in CA3, the region most damaged after intra-amygdala KA injection [36], showed only a modest increase 8 h after *status epilepticus* (Fig. 1e and f). The pattern of GFP staining later changed dramatically, becoming diffuse throughout the hippocampus by 24 h and no longer showing distinct borders around neuronal populations (Fig. 1e). Thus, UPS inhibition seems to be most prominent in hippocampal subfields spared from seizure-induced cell death and appears to progress through different hippocampal subfields involving different cell types over time following *status epilepticus*.

**Fig. 2:**
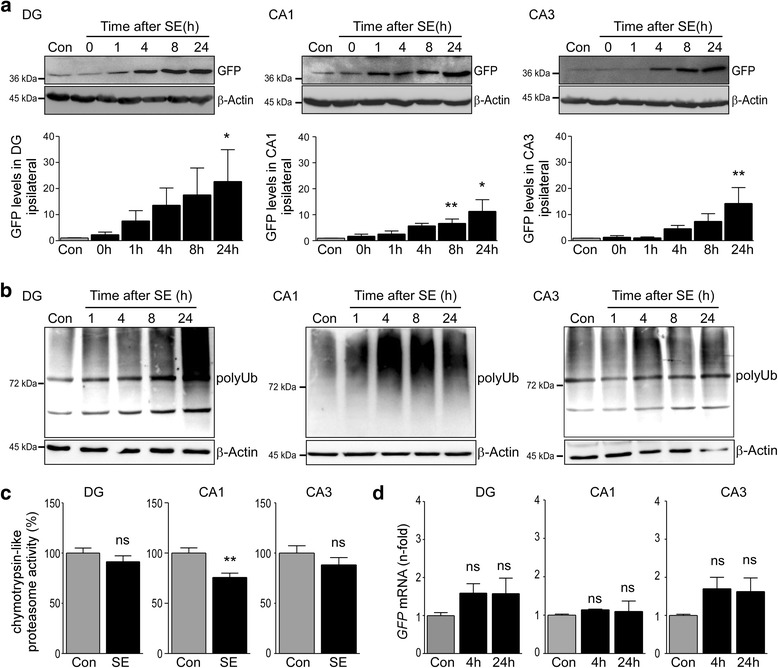
Subfield-specific UPS impairment after *status epilepticus* in the hippocampus. **a**. Representative Western (*n* = 1 per lane) and graphs showing increased GFP protein in the different ipsilateral hippocampal subfields DG, CA1 and CA3 at different time-points following *status epilepticus* in Ub^G76V^-GFP mice (mean ± sem, *p <0.05 by one-way ANOVA with Fisher’s post hoc test; *n* = 4 (0 h), 5 (24 h) and 6 (Con, 1 h, 4 h and 8 h)). **b**. Representative Western (*n* = 1 per lane) showing increased polyubiquitination (FK2 antibody) in C57/Bl6 wild-type mice after *status epilepticus* which is most prominent in the DG and CA1 subfield. **c**. Graph showing decreased chymotrypsin-like proteasome activity in the CA1 subfield of C57/Bl6 wild-type mice 4 h after *status epilepticus* when compared to control-injected mice. No changes could be observed in the remaining subfields DG (*p* = 0.289) and CA3 (*p* = 0.291) at the same time-point post-*status epilepticus* (4 h) (mean ± sem, **p <0.01 by student’s two-tailed *t*-test; *n* = 8 (Con, Control) and 6 (SE, *status epilepticus*) per group). **d.** Graphs showing no significant changes in *GFP* mRNA levels 4 h and 8 h after *status epilepticus* for the ipsi-lateral hippocampal subfields DG, CA1 and CA3 (mean ± sem, by one-way ANOVA with Fisher’s post hoc test; *n* = 4 per group). ns = non-significant

Immunohistochemical findings were confirmed by Western blotting using microdissected hippocampal subfields from a separate set of Ub^G76V^-GFP mice subjected to *status epilepticus*. Consistent with the immunohistochemistry, GFP first increased in DG and CA1 8 h post-*status epilepticus* with the most prominent GFP increase in the DG (>20-fold) (Fig. 2a). At 24 h after *status epilepticus*, GFP signal was increased in all hippocampal subfields including CA3 (Fig. 2a).

To assure that the subfield-specific increase in Ub^G76V^-GFP protein in fact reflects a diminished UPS function, we analyzed microdissected hippocampal subfields from wild-type C57/Bl6 mice subjected to KA-induced *status epilepticus*. As shown before for Ub^G76V^-GFP mice, polyubiquitination levels were increased in all subfields with highest levels in the damage-protected hippocampal subfields DG and CA1 (Fig. 2b).

Glutamate-induced excitotoxicity has been shown to reduce proteasome activity [24]. To determine whether the seizure-induced UPS impairment is due to a decrease in proteasome activity, proteasome catalytic activity assays were performed. To rule out that intrinsic variations in proteasome activity are the cause of the observed subfield specific response to seizures, chymotrypsin-like activity was compared between the different hippocampal subfields CA1, DG and CA3 in naive C57/Bl6 wild-type mice. No differences between the different hippocampal subfields in chymotrypsin-like proteasome activity were observed (Additional file 1: Figure S2A). We then subjected wild-type mice to KA-induced *status epilepticus* and measured chymotrypsin-like proteasome activity in all different hippocampal subfields at different time-points post KA injection. No changes in chymotrypsin-like proteasome activity could be observed at the time of lorazepam injection, 40 min after intra-amygdala KA injection when analyzing all three hippocampal subfields (Additional file 1: Figure S2C); however, a small decrease in chymotrypsin-like proteasome activity was evident 4 h post *status epilepticus* in the CA1 subfield of the hippocampus (Fig. 2c). This was most likely due to changes in the catalytic activity of the 20S proteasome itself, as no changes in hippocampal 20S proteasome levels could be observed after *status epilepticus* (Additional file 1: Figure S2D). To test whether changes in GFP transcription might be the cause of increased GFP protein levels after *status epilepticus* we measured GFP transcripts at two different time-points in the hippocampal subfields DG, CA3 and CA1 (Fig. 2d). Only a minor, non-significant increase in *GFP* transcripts 4 h and 24 h post-*status epilepticus* could be observed which was present in all three hippocampal subfields (Fig. 2d). No differences could be observed in the magnitude of increase between the different hippocampal subfields, ruling out that increased *GFP* transcription accounts for the differences observed in GFP protein levels between hippocampal subfields (Fig. 2d).

### UPS impairment in the undamaged contralateral hippocampus after *status epilepticus*

**Fig. 3:**
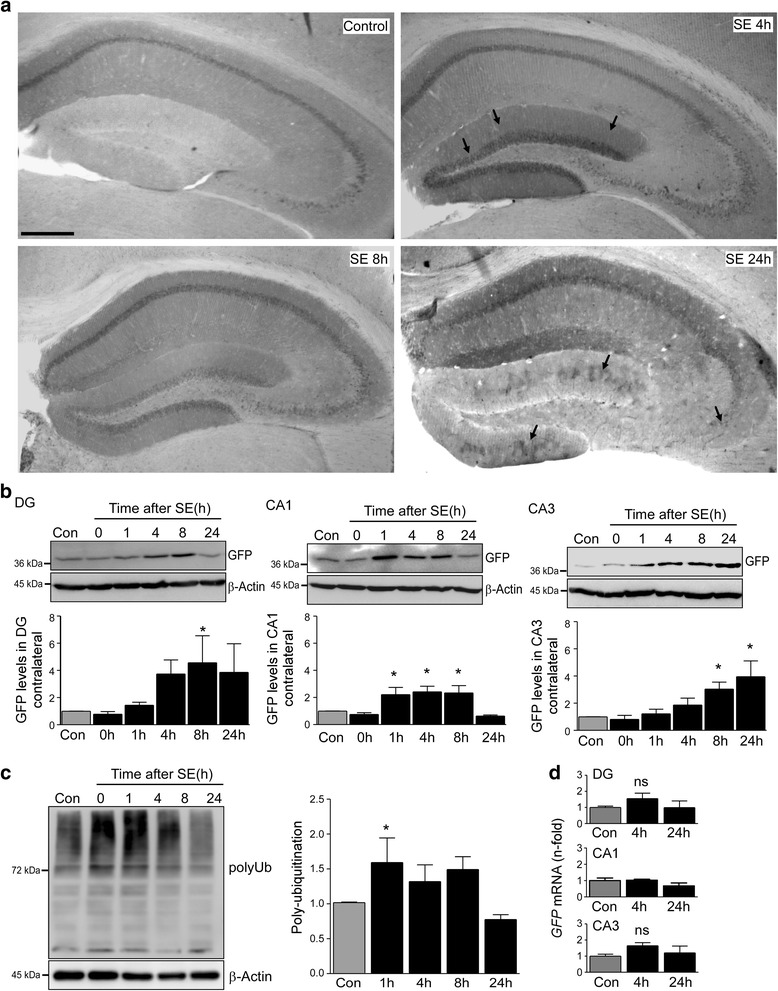
UPS impairment in the contralateral hippocampus after *status epilepticus*. **a**. Representative photomicrographs (5x lens) showing subfield-specific increased GFP-reporter accumulation in the contralateral hippocampus of transgenic Ub^G76V^-GFP mice subjected to intra-amygdala KA-induced *status epilepticus*. Note, similar GFP staining pattern as observed in the ipsilateral hippocampus. Scale bar, 500 μm. **b**. Representative Western (*n* = 1 per lane) and graphs showing increased GFP levels in the contralateral hippocampal subfields DG, CA1 and CA3 after *status epilepticus* (mean ± sem, *p <0.05 by one-way ANOVA with Fisher’s post hoc test; *n* = 4 per group). **c.** Representative Western (*n* = 1 per lane) and graph showing increased polyubiquitination levels (FK2 antibody) in the contralateral hippocampus after intra-amygdala KA induced-*status epilepticus* (mean ± sem, *p <0.05 by one-way ANOVA with Fisher’s post hoc test; *n* = 4 per group). **d.** Graphs showing no significant changes in *GFP* mRNA levels 4 h and 8 h after *status epilepticus* for the contralateral hippocampal subfields DG, CA1 and CA3 (mean ± sem, by one-way ANOVA with Fisher’s post hoc test; *n* = 4 per group). ns = non-significant

To obtain further evidence of proteasome inhibition being a characteristic of damage- resistant brain areas, we analysed the contralateral hippocampus of Ub^G76V^-GFP reporter mice subjected to intra-amygdala KA-induced *status epilepticus*. Previous studies have confirmed, while being protected from seizure-induced cell death, contralateral hippocampal structures are recruited during *status epilepticus*, however, to a lesser extent [40]. As previously shown in Fig. 1e, Ub^G76V^-GFP signal in the contralateral hippocampus was low in control vehicle-injected animals with highest levels observed in the CA1 subfield (Fig. 3a). However as seen before, GFP levels increased sharply in the contralateral hippocampus after *status epilepticus* (Fig. 3a and b). This increase was most evident in the DG (~5-fold) at early time-points. Scattered GFP staining throughout the contralateral hippocampus appeared 24 h after seizure suppression (Fig. 3a). Immunohistological results were supported by Western blotting against GFP using tissue extracts from the three contralateral hippocampal subfields DG, CA3 and CA1 (Fig. 3b). Western blotting using the polyubiquitination-recognizing antibody FK2 [41] confirmed increased polyubiquitination in the contralateral hippocampus post-*status epilepticus* (Fig. 3c). No changes in *GFP* mRNA levels could be observed for the different subfields ruling out increased *GFP* transcription being responsible for the increase in GFP protein (Fig. 3d). These results confirm UPS impairment as a global response in the brain to prolonged seizure activity.

### Cell-specific UPS impairment after *status epilepticus*

**Fig. 4:**
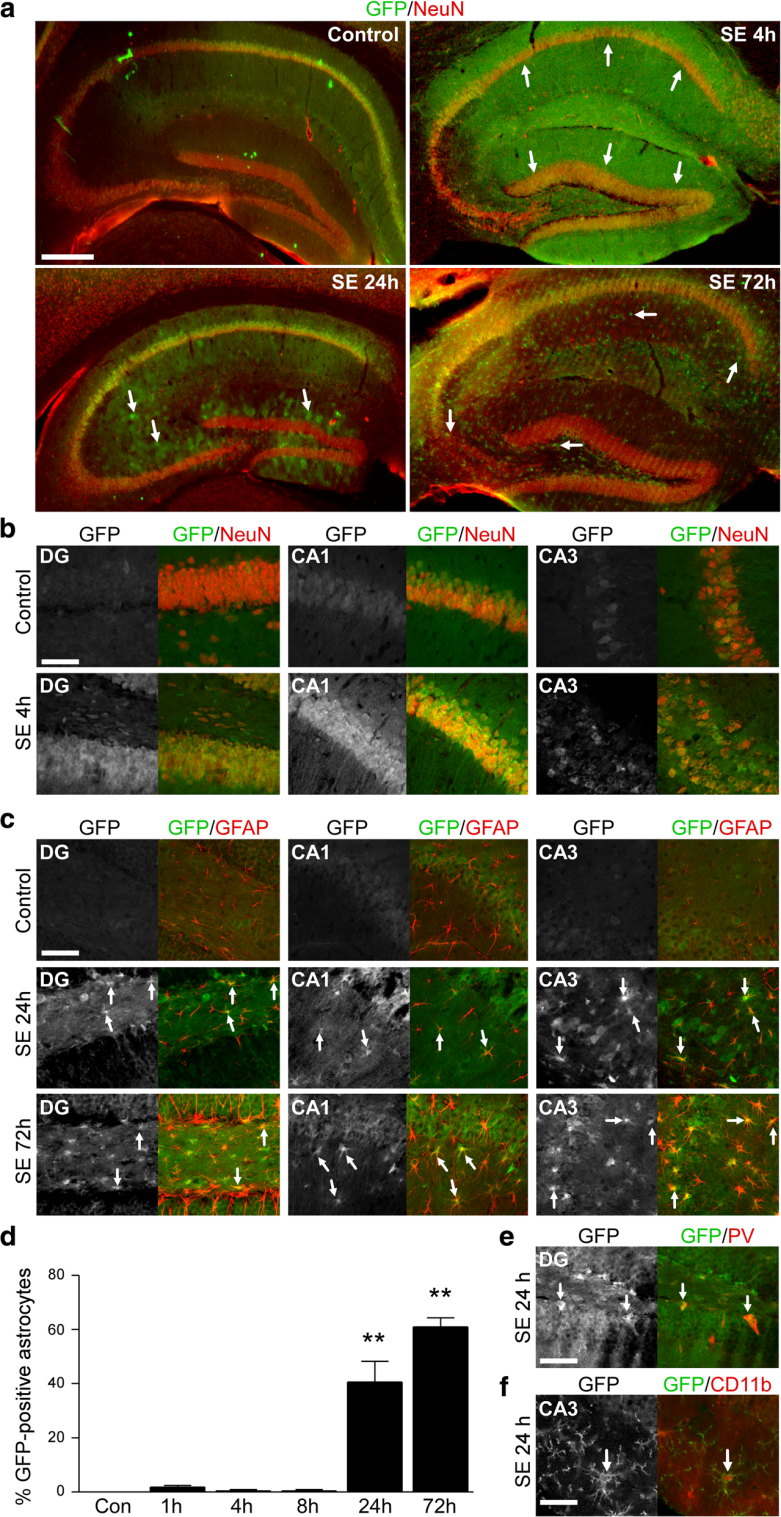
Cell-specific UPS impairment after *status epilepticus*. **a**. Representative photomicrographs (5x lens) of coronal cut hippocampal brain slices from Ub^G76V^-GFP reporter mice double stained against GFP (*green*) and the neuronal marker NeuN (*red*) at different time-points post-*status epilepticus*. Scale bar, 500 μm. **b**. Representative photomicrographs (40x lens) showing co-localization between the neuronal marker NeuN (*red*) and GFP (*green*) in the hippocampal subfields DG, CA1 and CA3 4 h post-*status epilepticus* in Ub^G76V^-GFP reporter mice. Scale bar, 50 μm. **c**. Representative photomicrographs (40x lens) showing co-localization between the astrocyte marker GFAP (*red*) and GFP (*green*) in the ipsilateral hippocampal subfields DG, CA1 and CA3 24 h and 72 h post-*status epilepticus* in Ub^G76V^-GFP reporter mice. Scale bar, 50 μm. **d**. Graph showing percentage of hippocampal astrocytes positive for GFP after the induction of *status epilepticus* (mean ± sem, **p <0.01 by one-way ANOVA with Fisher’s post hoc test; *n* = 4 per group). **e**. Representative photomicrograph (40 x lens) showing co-localization between GFP (*green*) and the inter-neuronal marker parvalbumin (PV) (*red*) in the hilar sub-region of the ipsilateral hippocampus of Ub^G76V^-GFP reporter mice 24 h post-*status epilepticus*. Scale bar, 50 μm. **f**. Representative photomicrograph (40 x lens) showing co-localization between GFP (*green*) and the microglial marker CD11b (*red*) in the CA3 ipsilateral hippocampal subfield of Ub^G76V^-GFP reporter mice 24 h post-*status epilepticus*. Scale bar, 50 μm

To identify the cell types in which UPS inhibition occurred, we performed immunofluorescence staining using antibodies against GFP and specific cell markers for neurons (NeuN), astrocytes (GFAP) and microglia (CD11b). Double-staining of GFP and NeuN revealed a mainly neuronal GFP accumulation at early time-points post-*status epilepticus* (Fig. 4a and b). Co-localization of GFP with the glial marker GFAP was essentially absent at these early time-points (Fig. 4d). However, co-localization with the astrocyte marker GFAP increased sharply at later stages after *status epilepticus* (24 h and 72 h post-*status epilepticus*) (Fig. 4c and d) with ~50% of GFAP-positive cells being positive for GFP (Fig. 4d). We also observed co-localization of GFP with the interneuron marker Parvalbumin (PV) (Fig. 4e) and occasional co-localization of GFP with the microglial marker CD11b at later time-points after *status epilepticus* (24 h) (Fig. 4f). These results suggest that *status epilepticus* leads to a cell-specific UPS impairment, switching from an early UPS impairment in neurons to appear at later stages in glial cells, in particular astrocytes.

### Proteasome inhibition in experimental epilepsy

**Fig. 5:**
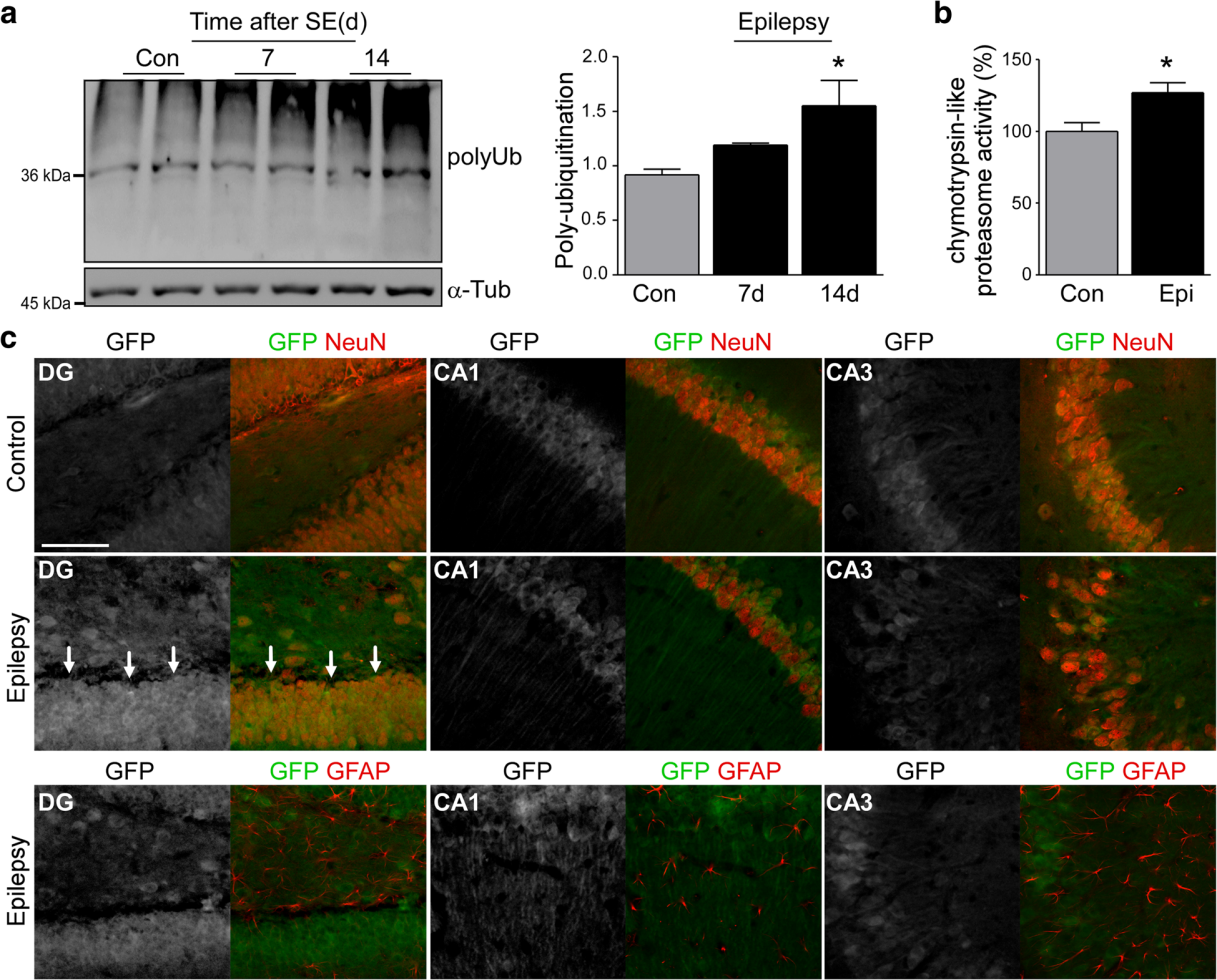
UPS impairment during epilepsy*.* **a**. Representative Western (*n* = 1 per lane) and graph showing increased polyubiquitination (FK2) in the ipsilateral hippocampus of epileptic C57/Bl6 wild-type mice 14 days post-intraamygdala KA (mean ± sem, *p <0.05 by one-way ANOVA with Fisher’s post hoc test; *n* = 4 per group). **b**. Increased chymotrypsin-like proteasome activity in the ipsilateral hippocampus of epileptic C57/Bl6 wild-type mice 14 days post-intraamygdala KA (mean ± sem, *p <0.05 by student’s two-tailed *t*-test; *n* = 4 per group). **c**. Representative photomicrograph (40 x lens) showing co-localization between GFP (*green*) and the neuronal marker NeuN (*red*) in the different hippocampal subfields of the ipsilateral hippocampus of Ub^G76V^-GFP reporter mice 14 days post-*status epilepticus* which was most evident in the DG (arrows). Co-localization of GFP with the astrocyte marker GFAP (*red*) in the same mice was mainly absent. Scale bar, 50 μm

Next, we explored whether UPS impairment also occurs once epilepsy is established. However, the lack of suitable control material (e.g. autopsy) prevented a full analysis of UPS changes in patient tissue (note: polyubiquitination levels rapidly decrease due to a post-mortem effect likely attributed to the depletion of ATP required for Ub activation [11]; Additional file 1: Figure S3A). Accordingly, we focused our analysis on tissue from the intra-amygdala KA model. In this model, all mice develop spontaneous recurrent seizures within three to five days post-*status epilepticus* with animals typically experiencing one to four epileptic seizures per day [36]. Western blot analysis using the polyubiquitinated substrate recognizing antibody FK2 revealed an increase in hippocampal polyubiquitination levels in samples from epileptic mice at day 14-post KA injection (Fig. 5a). To test whether the accumulation of polyubiquitinated conjugates is accompanied by reduced proteasome activity, chymotrypsin-like proteasome activity was assessed in hippocampal lysates from epileptic C57/Bl6 wild-type mice. Unexpectedly, and in contrast to what was observed after *status epilepticus*, hippocampal tissue from epileptic mice showed increased chymotrypsin-like activity (Fig. 5b). However, this may have been due to increased expression levels of 20S proteasome subunits (Additional file 1: Figure S3B).

To identify cell types experiencing UPS inhibition in epileptic mice, we analyzed tissue sections from Ub^G76V^-GFP reporter mice 14 days after *status epilepticus*. Here, neurons of the DG were the main cell-population displaying increased GFP levels (Fig. 5c). At this time-point, co-localization between GFP and GFAP-positive astrocytes was almost absent (Fig. 5c).

### Proteasome inhibition can protect damage-vulnerable hippocampal subfields against seizure-induced cell death

**Fig. 6:**
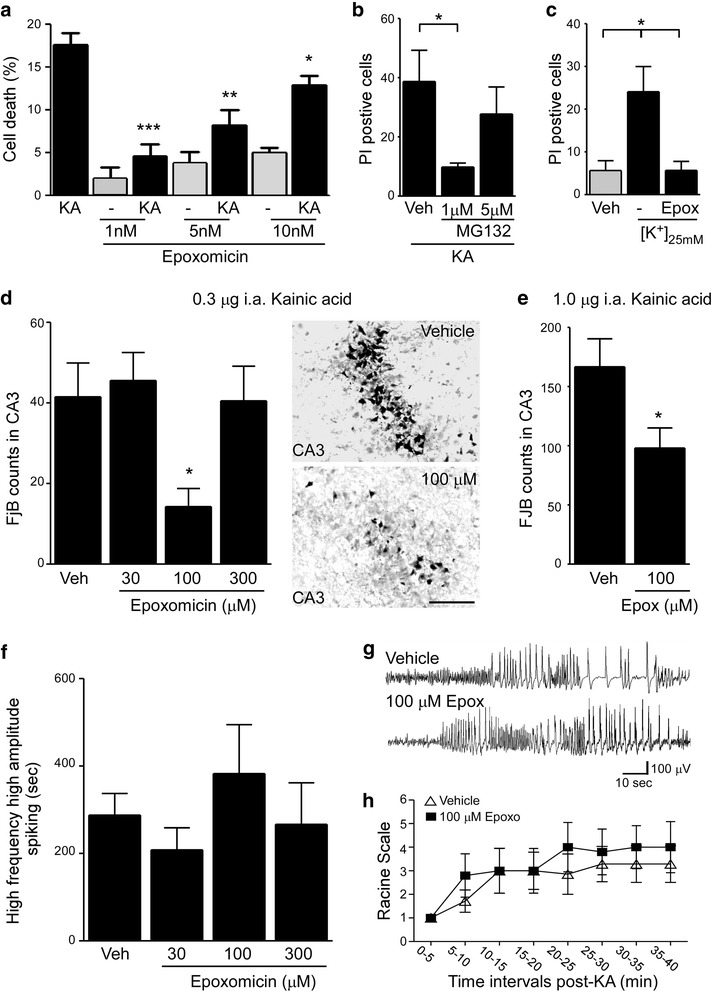
Proteasome inhibition protects from seizure-induced cell death. **a** Graph showing decreased KA-induced cell death in hippocampal primary neurons when pre-treated with the specific proteasome inhibitor epoxomicin (mean ± sem, *p <0.05, **p <0.01 and ***p <0.001 by one-way ANOVA with Fisher’s post hoc test; *n* = 3 per group). **b.** Decreased KA-induced cell death in hippocampal primary neurons when pre-treated with the specific proteasome inhibitor MG132 (mean ± sem, *p <0.05 by one-way ANOVA with Fisher’s post hoc test; *n* = 4 per group). **c**. Epoxomicin pre-treatment protects against cell death caused by increased extracellular [K^+^] (25 mM) in primary hippocampal cultures (mean ± sem, *p <0.05 by one way ANOVA with Fisher’s post hoc test; *n* = 3 per group). **d.** Graph and representative images of the CA3 region of the ipsilateral hippocampus showing decreased seizure-induced cell death in mice pretreated with epoxomicin. *Status epilepticus* was induced by an intra-amygdala injection of 0.3 μg KA and neuronal cell death was assessed 24 h later by counting FjB-positive cells in the ipsilateral hippocampal subfield CA3 (mean ± sem, *p <0.05 by one-way ANOVA with Fisher’s post hoc test; *n* = 11 (Control), 5 (30 and 300 μM epoxomicin) and 7 (100 μM epoxomicin). Scale bar, 50 μm. **e.** Pre-treatment with epoxomicin protects against seizure-induced cell death in a model of predominant necrotic cell death. Here, *status epilepticus* was induced by an intra-amygdala injection of 1.0 μg KA and neurodegeneration analyzed by counting FjB-positive cells in the hippocampal subfield CA3 24 h post-KA injection (mean ± sem, *p <0.05 by student’s two-tailed *t*-test; *n* = 4 (Control) and 5 (100 μM epoxomicin). **f.** Similar amount of high amplitude and high frequency polyspiking in mice subjected to *status epilepticus* during a recording period of 40 min pre-treated with different concentrations of epoxomicin (analysis started at time-point of intra-amygdala KA (0.3 μg) injection until lorazepam administration) (mean ± sem, *p <0.05 by ANOVA with Fisher’s post hoc test; *n* = 8 (Control), 5 (30 μM), 7 (100 μM) and 6 (300 μM)). **g.** Representative electroencephalogram (EEG) traces during *status epilepticus* from mice treated with vehicle or 100 μM epoxomicin showing similar high frequency and high amplitude polyspiking. **h.** Graph showing no significant differences in clinical seizures during the time of intra-amygdala KA (0.3 μg) injection until lorazepam administration between mice pre-treated with vehicle or 100 μM epoxomicin (mean ± sem, by two-way ANOVA with Bonferroni post hoc test; *n* = 5 per group)

To establish whether proteasome inhibition can protect neurons from excitotoxicity, primary hippocampal neurons were treated with different concentrations of two different proteasome inhibitors, epoxomicin [42] and MG132 [43] before being exposed to a cell death-causing concentration of KA (0.3 μM) [44]. Consistent with proteasome inhibition being protective, epoxomicin pre-treated hippocampal neurons showed significantly less cell death when compared to vehicle-treated primary hippocampal neurons (Fig. 6a). Protection was most obvious at lower doses of epoxomicin (1 nM). The protection still persisted at higher doses of epoxomicin (10 nM), however, to a lesser extent. This was possibly due to neurotoxic effects of proteasome inhibition [8, 45] (Fig. 6a). To test whether protection is specific to epoxomicin, we used MG132, which belongs to a different class of proteasome inhibitors [43]. As observed for epoxomicin, MG132 treatment protected neurons from cell death (Fig. 6b). Again, this neuroprotective effect was more pronounced at lower concentrations of MG132 (Fig. 6b) mirroring the effect of epoxomicin. Finally, to show that this protection was not specific to the KA in vitro model, primary hippocampal neurons were treated with a cell death-causing dose of potassium (25 mM). As observed before with KA, pre-treatment with epoxomicin protected against potassium-induced cell death in vitro (Fig. 6c).

Next, to explore whether proteasome inhibition is neuroprotective in vivo against *status epilepticus*, C57/Bl6 wild-type mice were pre-treated with the proteasome inhibitor epoxomicin 30 min before the injection of KA. Proteasome activity assays, Western blotting and immunostaining against GFP confirmed hippocampal UPS inhibition after intracerebroventricular (i.c.v.) delivery of epoxomicin (Additional file 1: Figure S4A - C). Mice which received 100 μM i.c.v. epoxomicin prior to intra-amygdala KA injection displayed significantly less neurodegeneration in the ipsilateral hippocampal subfield CA3 when compared to vehicle-injected mice as assessed by FjB staining 24 h after *status epilepticus* (Fig. 6d). To test whether a more severe seizure model, with more extensive CA3 damage, would alter the protective effects of epoxomicin, we used a second model of *status epilepticus* displaying predominant necrotic cell death [46]. As observed before, epoxomicin-treated mice displayed less cell death in the ipsilateral CA3 after *status epilepticus* when compared to vehicle-injected mice (Fig. 6e) further confirming the neuroprotective potential of proteasome inhibition against seizure-induced cell death. Finally, to rule out any possible effects of differences in seizure severity on seizure-induced cell death, we analyzed high amplitude high frequency discharges, synonymous with injury-causing electrographic activity [47], during the time from KA injection until the administration of the anticonvulsant lorazepam. All treatment groups underwent similar seizure severity and seizure-induced behaviour changes (Fig. 6f-h) suggesting neuroprotection was not secondary to an anticonvulsant effect of epoxomicin.

## Discussion

In the present study we show an increased accumulation of polyubiquitinated proteins in the hippocampus after prolonged seizures and during epilepsy suggesting a seizure-induced inhibition of the UPS. Most strikingly and unexpectedly, seizure-induced UPS impairment was mainly evident in brain areas resistant to seizure-induced cell death including neurons and astrocytes. In line with a neuroprotective role of proteasome inhibition during seizures, mice treated with the specific proteasome inhibitor epoxomicin displayed less neurodegeneration. These findings support proteasome inhibition as an endogenous protective mechanism against seizure-induced cell death.

Despite differences in disease etiology (e.g. β-amyloid, polyglutamine expansion, neurodevelopmental abnormalities and other) several chronic brain disorders share common clinical symptoms (e.g. cognitive deficits, seizures and psychological problems such as depression and anxiety) implying the emerging concept of existing shared pathological pathway activation among different brain diseases [48–50]. Proteasome inhibition and the accumulation of polyubiquitinated aggregates are a common characteristic of chronic neurodegenerative diseases such as Alzheimer’s and Huntington’s [9, 51] and an impairment of the UPS has also been reported after acute insults to the brain such as stroke and traumatic brain injury [10, 52]. Likewise, emerging evidence suggests a dysfunction of the UPS in neurological diseases such as epilepsy [29]. However, previous studies lacked evidence showing a direct link between seizures and proteasome inhibition and whether seizures lead to an inhibition of the proteasome in vivo has not been fully established. Studies were limited to determining the expression levels of specific subunits of the immunoproteasome [28, 29] or to identify the impact certain mutations of genes involved in UPS functions have on pathology (e.g. different E3 ligases, deubiquitinating proteins) [26, 31]. Recent in vitro experiments have suggested that glutamate-induced excitotoxicity may lead to UPS inhibition mediated by NMDA receptor activation [24]. We extend these studies here by showing that prolonged seizures lead to an impairment of the UPS with the subsequent accumulation of polyubiquitinated substrates in the brain. We do not know what causes an inhibition of the proteasome after seizures. In line with in vitro results [24], we have observed a down-regulation of chymotrypsin-like activity in the hippocampal subfield CA1 after KA-induced seizures. This may contribute to the accumulation of polyubiquitinated proteins in the hippocampus after seizures. However, the hippocampal subfield showing the strongest increase of Ub^G76V^-GFP reporter protein levels and polyubiquitinated conjugates after *status epilepticus* in our model was the DG subfield which showed only a marginal inhibition of the catalytic activity of the 20S proteasome. Previous studies have shown a dose-dependent increase in Ub^G76V^-GFP reporter in hippocampal neurons treated with epoxomicin [35], therefore, our results suggest that other mechanisms may contribute to proteasome inhibition. Moreover, no differences in 20S proteasome activity was observed at the time of lorazepam administration, further suggesting only a small contribution of the catalytic down-regulation of the 20S proteasome to UPS impairment. Other factors may contribute to the accumulation of polyubiquitinated proteins after prolonged seizures. *Status epilepticus* leads to a depletion of intracellular ATP [53], possibly impeding E1-mediated Ub activation and compromising a correct functioning of the ATP-consuming 20S proteasome [11]. Increased intracellular Ca^2+^ during excitotoxicity might also lead to a dysfunction of the proteasome [24]; however, other reports have suggested that increased intracellular Ca^2+^ levels lead to increased proteasome activity [54]. Furthermore, s*tatus epilepticus* leads to an increase in ER-stress [55] and an impairment of autophagy [56], a second major intracellular protein-degrading mechanism, thereby potentially leading to an overload of substrates which exceeds the capacity of the proteasome. Regardless of the mechanism, our results show that seizures lead to an impairment of the UPS with the subsequent accumulation of poly-ubiquitinated substrates in the hippocampus. Interestingly, seizures are a common co-morbidity of many neurodegenerative diseases such as Alzheimer’s and Huntington’s [57, 58] possibly contributing to the accumulation of polyubiquitinated aggregates during these diseases.

Our second major finding was that proteasome inhibition was most evident in hippocampal subfields resistant to seizure-induced cell death. This was unexpected since proteasome inhibition is typically associated with a failure of homeostasis and a driver of neurodegeneration [8]. We do not know why UPS impairment was most evident in these brain regions. However, comparing GFP increases between both brain hemispheres and the different hippocampal subfields, it is tempting to speculate that the level of proteasome inhibition reflects seizure progression throughout the ipsilateral and contralateral hippocampus [40] with the DG being the major site of excitatory input into the hippocampus [59]. While proteasome inhibition was most apparent in neurons at early stages after *status epilepticus*, astrocytes were the main cell population affected later by UPS impairment. This might be due to the fact that neurons are most likely the cell population activated during the early stages of seizure-induced pathology, while glial responses occur at later stages [60]. Interestingly, proteasome inhibition has been shown to decrease astrogliosis in vitro and in vivo [61]. Furthermore, recent studies demonstrated that astrogliosis alone, in the absence of any other pathology, is sufficient to cause epilepsy in mice [62, 63]. This may suggest a potential anti-epileptic effect provided by proteasome inhibition in glia. UPS impairment in astrocytes is not unique to our seizure model and has been described for other diseases including Huntington’s disease [64]; amyotrophic lateral sclerosis [65] and prion disease [39]. Moreover, astrocytes seem to be well adapted to support long-lasting UPS impairment probably due to the efficient induction of heat shock responses [66].

Why does the ipsilateral CA3 subfield of the hippocampus display a delayed inhibition of the UPS? The CA3 subfield is particularly vulnerable in our model [36] possibly due to the high density of kainate receptors [67]. Although a NMDA-mediated down-regulation of the UPS has been observed after excitotoxicity [24], this has been restricted to the nuclear compartment [24]. On the other hand, increased intracellular Ca^2+^ concentrations have been shown to activate the proteasome [54] which may delay the accumulation of polyubiquitinated conjugates at early time-points after *status epilepticus*. Another possible explanation for the delayed accumulation of polyubiquitinated proteins in CA3 may be increased inhibition of translation after seizures [68] leading to fewer substrates to be degraded by the proteasome.

Although the spatio-temporal profile of UPS inhibition has not been reported previously after *status epilepticus*, there have been numerous reports of UPS inhibition after ischemic brain injury [27, 69, 70]. Here, proteasome inhibition was most evident in the hippocampal subfield CA1, a brain region vulnerable to ischemia-induced cell death, whereas only a transient polyubiquitination could be observed in the remaining cell-death resistant hippocampal subfields CA3 and DG [27]. This is different to *status epilepticus*, where, in contrast to ischemia, cell death-resistant brain regions showed the highest increase in polyubiquitination levels, suggesting a unique pathology according to brain insult. Interestingly, during ischemia the hippocampal subfield CA1 showed the strongest reduction in the catalytic activity of the proteasome [71], similar to findings in our mouse model of *status epilepticus*, suggesting that some similarities may exist between different conditions. We do not know why the hippocampal subfield CA1 is more prone to a reduction in proteasome activity after seizures and why this downregulation is not accompanied by a bigger increase in polyubiquitinated proteins after *status epilepticus* when compared to the other hippocampal subfields. Differences in glutamate receptor recruitment during *status epilepticus* (e.g. NMDA subtype vs. kainic acid or AMPA receptor) with the resulting differences in intracellular calcium levels, may affect proteasome activity [24, 54]. Differences in the subfield-specific clearance of damaged proteins and/or ER-stress during *status epilepticus* might also contribute to differences in the accumulation of polyubiquitinated proteins.

Why does UPS impairment protect against seizure-induced cell death? The protection is presumably a result of an accumulation of proteins that would otherwise be degraded. However, we do not know their identity. The most likely candidates probably include proteins involved in inflammatory processes such as NFκB which has been shown to mediate proteasome inhibitor-mediated neuroprotection after ischemia [72, 73]. Other proteins such as heat shock proteins [74] or synaptic proteins may also be involved. This could be assessed in the future using Tandem mass spectrometry (MS-MS)-type approaches. Another option may be that accumulated proteins interrupt signalling pathways that would normally be able to progress through to kill neurons or, alternatively, the inhibition of the ATP-consuming UPS may help protect against excessive depletion of energy reserves during seizures.

Emerging evidence suggests that the UPS controls neurotransmission by regulating crucial processes such as synaptic plasticity, spinogenesis and levels of functional neurotransmitter receptors [17]. We have not observed any changes in seizure severity during *status epilepticus* after epoxomicin treatment. However, a potential impact of the proteasome on seizure generation or seizure severity might be more important under chronic conditions. Epileptic mice in our study showed increased polyubiquitination levels in the hippocampus suggesting a chronic impairment of the UPS which may contribute to hyperexcitability. In line with this, recent data has shown a more severe epileptic phenotype in rats treated with the proteasome inhibitor MG-132 [75], which, in contrast to epoxomicin, also inhibits calpain activity [76]. While acute/transient UPS inhibition may be protective, long-lasting inhibition may be neurotoxic. Indeed, inhibition of the UPS has been reported to elicit neurodegeneration directly [8, 45]. Therefore, it is unlikely proteasome inhibitors will be used as neuroprotective or anti-epileptic drugs. However, they provide important insight into molecular pathogenesis and, if we can identify the affected pathways/proteins involved, this may lead to novel approaches for the treatment of *status epilepticus* and epilepsy.

## Conclusions

Our data show that prolonged seizures lead to an impairment of the UPS which is most prominent in brain areas resistant to seizure-induced cell death suggesting proteasome inhibition as an endogenous protective mechanism.

## Methods

### Mouse seizure models

For our studies we used adult male mice (20–25 g) (C57Bl/6 (Harlan laboratories, Bicester, UK)) and mice ubiquitously expressing the ubiquitin fusion degradation substrate ubiquitin^G76V^-green fluorescent protein (Ub^G76V^-GFP) [38]. The main experiments used the intra-amygdala KA-induced *status epilepticus* [36]. Briefly, mice were anesthetized using isoflurane (3–5%) and maintained normothermic by means of a feedback-controlled heat blanket (Harvard Apparatus Ltd, Kent, England). Then, mice were placed in a stereotaxic frame and following a midline scalp incision, three partial craniotomies were performed. Surface EEG was recorded from three scull-mounted recording electrodes (Bilaney Consultants Ltd, Sevenoaks, UK) placed above the dorsal hippocampus and frontal cortex. EEG was recorded using a Grass Comet digital EEG (Medivent Ltd, Lucan, Ireland). A guide cannula was affixed over the dura (coordinates from Bregma: AP = −0.94; L = −2.85 mm) and the entire skull assembly fixed in place with dental cement. Anaesthesia was discontinued, EEG recordings were commenced, and then a 31-gauge internal cannula (Bilaney Consultants Ltd, Sevenoaks, UK) was inserted into the lumen of the guide to inject KA (Sigma-Aldrich, Dublin, Ireland) (0.3 μg in 0.2 μl of vehicle or 1.0 μg in 0.2 μl of vehicle; phosphate-buffered saline (PBS), pH adjusted to 7.4) into the amygdala. Non-seizure control animals received the same volume of intra-amygdala vehicle. EEG was recorded until intra-peritoneal lorazepam (6 mg/kg) administration at 40 min post-KA injection. In a subgroup of mice, *status epilepticus* was induced by a subcutaneous injection of pilocarpine (Sigma-Aldrich, Dublin, Ireland) at 340 mg/kg body weight, 20 min after injection of methyl-scopolamine (1 mg/kg) (Sigma-Aldrich, Dublin, Ireland) [40].

Mice were euthanized at different time-points post lorazepam administration (0, 1, 4, 8, 24 and 72 h and 7 and 14 days) by transcardial perfusion with PBS or 4% paraformaldehyde (PFA) or brains were microdissected on ice and processed for protein analysis.

### Quantification of EEG and behaviour assessment of seizure severity

Cortical EEG recordings were analyzed off-line using manual assessment as described [77]. The duration of high-frequency (>5 Hz) and high-amplitude (>2X baseline) polyspike discharges of ≥5 s duration (HAHFDs) which are synonymous with injury-causing electrographic activity [47] was counted by a reviewer blind to treatment. Changes in seizure-induced behaviour were scored according to a modified Racine Scale as reported previously [78]. Score 1, immobility and freezing; Score 2, forelimb and or tail extension, rigid posture; 3, repetitive movements, head bobbing; Score 4, rearing and falling; Score 5, continuous rearing and falling; Score 6, severe tonic–clonic seizures. Mice were scored every 5 min for 40 min after KA injection. The highest score attained during each 5 min period was recorded by an observer blinded to treatment.

### In vivo drug treatments

Drugs were delivered via intracerebroventricular (i.c.v.) injections as described before [77] (coordinates for i.c.v. injections were Bregma: AP = −0.3 mm, L = −1.0 mm, V = −2.0 mm). Mice received a 2 μl infusion of the specific proteasome inhibitor epoxomicin (Sigma-Aldrich, Dublin, Ireland) at 30 μM, 100 μM or 300 μM (diluted in 100% dimethyl sulphoxide, (Sigma-Aldrich, Dublin, Ireland) or vehicle 30 min before the induction of *status epilepticus* and 60 min after KA injection.

### Proteasome activity assay

To measure chymotrypsin-like proteasome activity, we used the fluorigenic peptide N-succinyl-Leu-Leu-Val-Tyr-7-amino-4-methyl-coumarin (suc-LLVYAMC) (Calbiochem, Nottingham, UK). Tissue or cells were homogenised in lysis buffer (10 mM HEPES, 42 mM KCl, 5 mM MgCl2, 0.1 mM EDTA, 0.1 mM EGTA, 1 mM DTT, 0.5% (w/v) CHAPS). Then, lysates were incubated with reaction buffer (25 mM HEPES, pH 7.4, 0.5 mM EDTA, pH 8) containing suc-LLVY-AMC. Using 360 nm excitation, fluorescence was measured at 465 nm and 37 °C using a plate reader with the appropriate filters (GENios, Tecan, Weymouth, UK). Fluorescence signals were normalized to the protein concentrations, which were determined by Bradford assay (Thermo Fisher Scientific, Dublin, Ireland).

### Western blotting

Western blotting was performed as previously described [40]. Whole hippocampi or hippocampal subfields (dentate gyrus (DG), CA1 and CA3) were homogenized in lysis buffer and protein concentration was determined. 30 μg protein samples were boiled in gel-loading buffer and separated on 10% to 12% SDS-PAGE gels. Proteins were transferred onto nitrocellulose membranes (BioRad, Hercules, USA) and then incubated with antibodies against the following: GFP (1/500, Cell Biolabs, CA, USA, AKR/020), FK-2 (polyubiquitin) (1/1000, Merck Millipore, Billerica, MA, U.S.A), C-Fos (1/400, Santa Cruz Biotechnology, CA, U.S.A), Proteasome 20S (1/1000, Enzo Life Science, Exeter, UK), β-Actin (1/1000, Sigma-Aldrich, Dublin, Ireland) and α-Tubulin (1/1000, Santa Cruz Biotechnology, Heidelberg, Germany). Membranes were then incubated with horseradish peroxidase-conjugated secondary antibodies (Jackson Immuno Research, Plymouth, PA) and bands visualized using Supersignal West Pico Chemiluminescent Substrate (Pierce, Rockford, IL). Images were captured using a Fuji-film LAS-300, densitometry performed using AlphaEaseFC4.0 software and data expressed as change relative to control.

### RNA extraction and real time PCR

Total mRNA was extracted as previously described using the Trizol protocol [40]. Quantity of mRNA was measured using a Nanodrop Spectrophotometer (Thermo Fisher Scientific, Rockford, IL, USA) and RNA dilutions were made up in nuclease-free water. For analysis of mRNA expression, 1 μg of total RNA was used to generate cDNA by reverse transcription using a Superscript II Reverse Transcriptase enzyme (Thermo-Fisher, MA, USA). Quantitative real-time PCR was performed using a LightCycler 1.5 (Roche Diagnostics GmbH, Mannheim, Germany) in combination with QuantiTech SYBR Green PCR kit (Qiagen, Hilden, Germany) as per the manufacturer’s protocol and 1.25 μM of primer pair used. Data were represented as 2^−ΔΔCT^ and normalized to the expression of β-actin. Primers used: *GFP* (F: acgtaaacggccacaacttc, R: aagtcgtgctgcttcatgtg); *β-actin* (F: gggtgtgatggtgggaatgg, R: ggttggccttagggttcagg).

### Histopathology

Neuronal damage was assessed by FjB (Merck Millipore, CA, USA) as described before [77]. Briefly, fresh-frozen coronal brain sections at the level of the dorsal hippocampus were post-fixed in formalin for 30 min, then incubated in 0.0006% potassium permanganate, washed and incubated for 20 min with a 0.001% FJB solution (Chemicon Europe Ltd., Chandlers Ford, UK). Once washed and dried, slides were coverslipped with DPX mounting solution (Sigma-Aldrich, Dublin, Ireland). Counts were the average of two adjacent sections assessed by an observer blinded to treatment.

### Immunofluorescence staining and confocal microcopy

For confocal microscopy, animals were transcardially perfused with 4% PFA, post-fixed and embedded in 2% agarose before sectioning on a vibratome (Leica Biosystems, Wetzlar, Germany). Sections were rinsed and treated with PBS containing 0.1% Triton^™^ X-100 and 1% foetal calf serum followed by incubation with the primary antibodies; GFP (1/500, Life Technologies, Thermo Fisher Scientific, Rockford, USA, A11122), NeuN (1/400; Merck Millipore, Billerica, MA, U.S.A), GFAP (1/500, Santa Cruz Biotechnology, CA, U.S.A), Cd11b (1/400, Abcam, Cambridge, UK) and PV (1/5000, Swant Inc, Marly, Switzerland). Sections were washed again and incubated with secondary antibodies coupled to Alexa Fluor® 488 or Alexa Fluor® 568 (BioSciences Ltd, Dublin, Ireland). Sections were then coverslipped with Fluorosave^™^ (Merck Millipore, Billerica, MA, U.S.A). Confocal images were acquired with a Leica TCR 6500 microscope equipped with four laser lines (405, 488, 561 and 653 nm) using a × 40 immersion oil objective.

To determine the percentage of GFP-positive astrocytes, sections were double-stained with antibodies detecting GFP and the astrocyte marker GFAP. 6 pictures were taken from each hippocampus (2 from each hippocampal subfield) at 20x magnification and astrocytes positive for GFAP alone or colocalized with GFP were counted in sections from control mice and at different timepoints post *status epilepticus* (1 h, 4 h, 8 h, 24 h and 72 h after KA injection). Images were analyzed using ImageJ.

### Diaminobenzidine (DAB) immunohistochemistry

DAB staining was performed as described before [77]. Briefly, mice were anesthetized and transcardially perfused with 4% PFA. Brains were post-fixed in 4% PFA and prepared for sectioning on vibratome as described. Next, 30 μm sagital brain sections were pretreated for 1 h with 1% BSA, 5% FBS, and 0.2% Triton X-100, and then incubated with the primary antibody GFP (1/500, Life Technologies, Thermo Fisher Scientific, Rockford, USA, A11122). Finally, brain sections were incubated in avidin–biotin complex using the Elite Vectastain kit (Vector Laboratories, Burlingame, CA, U.S.A). Chromogen reactions were performed with diaminobenzidine (Sigma-Aldrich, Dublin, Ireland) and 0.003% H_2_O_2_ for 10 min and, once dried, sections were coverslipped with Fluorosave.

### Primary hippocampal cell culture

Primary cultures of hippocampal neurons were prepared from E18.5 embryonic C57/Bl6 wild-type mice as described previously [40]. Briefly, neurons were plated onto poly-L-lysine and laminin bed and maintained in Neurobasal medium supplemented with B-27 and N2 (Biosciences, Belgium), at 37 °C in a humidified atmosphere with 5% (v/v) CO_2_ for 8 days. On in vitro day 8, cells were pre-treated with different doses of epoxomicin (1, 5 and 10 nM) or MG132 (Sigma-Aldrich, Dublin, Ireland) (1 and 5 μM) for 12 h. Cells were then treated with 0.3 μM KA or 25 mM KCl for 6 h. Cell death was determined using Propidium iodide (PI) (Sigma-Aldrich, MO, USA) staining. Cells were stained with PI and Hoechst and a minimum of three pictures were taken per experiment (20 x lens) blinded to treatment. Cell death was calculated as percentage of PI uptake in relation to Hoechst-positive cells. Each experiment was obtained from an independent dam (*n* = 3).

### Simulated post-mortem delay

To determine possible changes in UPS activity due to post-mortem delay differences, hippocampi were extracted from mice (adult C57Bl/6) after deep anesthesia with pentobarbital and decapitation. Hippocampi were either frozen immediately (‘surgical’ control) or frozen 4 h or 8 h after being left at room temperature (simulated post-mortem interval). Samples were then processed for Western blotting as described before [77].

### Data analysis

All data are presented as mean ± standard error of the mean. Two group comparisons were made using unpaired Student’s two-tailed *t*-test, while multi-group comparisons were made using one-way or two-way analysis of variance (ANOVA) followed by post hoc testing using Fisher’s exact test (StatView). Significance was accepted at *P* <0.05.

## Additional file

Additional file 1: Supplemental Figures S1-S4 (DOC 604 kb)

## Abbreviations

CA1/3: Cornu ammonis1/3
DG: Dentate gyrus
EEG: Electroencephalogram
FjB: Fluoro-Jade B
GFAP: Glial fibrillary acidic protein
GFP: Green fluorescent protein
KA: Kainic acid
PV: Parvalbumin
TLE: Temporal lobe epilepsy
Ub: Ubiquitin
Ub^G76V^-GFP: Ubiquitin^G76V^-green fluorescent protein
UPS: Ubiquitin proteasome system
