# Supplementary material for: Spatiotemporal progression of ubiquitin-proteasome system inhibition after status epilepticus suggests protective adaptation against hippocampal injury
Source: Mol Neurodegener. 2017 Feb 24;12:21. doi: 10.1186/s13024-017-0163-2 (PMC5324261; doi:10.1186/s13024-017-0163-2)
Supplement: Additional file 1: — Supplemental Figures S1-S4 (DOC 604 kb) [file 13024_2017_163_MOESM1_ESM.doc]

**Additional file 1:**

**Supplemental Figure 1**

**Recruitment of the ipsilateral hippocampus during status epilepticus**

To demonstrate a recruitment of the ipsi-lateral hippocampus during status epilepticus, Western blotting using hippocampal tissue from the ipsilateral side against the activity-regulated gene C-Fos was carried at different time-points post-intraamygdala KA injection. As expected C-Fos levels were significantly increased (**Fig. S1A**).


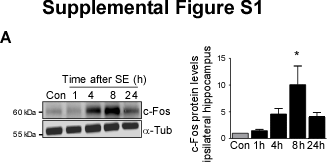


**Fig. S1.** Increased neuronal activity in the ipsi-lateral hippocampus after status epilepticus **(A)** Representative Western (n = 1 per lane) and graph showing increased protein levels of the neuronal activity-regulated gene *c-Fos* in the ipsilateral hippocampus after the induction of *status epilepticus* when compared to vehicle-injected control mice (mean ± sem, *p<0.05 by one-way ANOVA with Fischer’s post hoc test; n = 3 per group).

**Supplemental Figure 2**

**No changes in proteasome activity between different hippocampal subfields and at the time-point of seizure suppression**

To determine possible differences in proteasome activity between the three hippocampal subfields CA1, CA3 and DG, we carried out chymotrypsin-like proteasome activity assays in hippocampal tissue from naïve mice. Furthermore, the hippocampal subfield-specific induction of GFP was also measured. No differences in chymotrypsin-like proteasome activity and no differences in the induction of *GFP* mRNA between subfields could be observed in naïve mice (**Fig. S2A,B**). We also measured chymotrypsin-like proteasome activity in mice subjected to status epilepticus. No difference in chymotrypsin-like proteasome activity could be observed at the time-point of lorazepam injection (40 min-post-KA injection) (**Fig. S2C**). Ruling out differences in total proteasome amount, 20S subunit expression levels of the proteasome remained unchanged after status epilepticus (**Fig. S2D**).


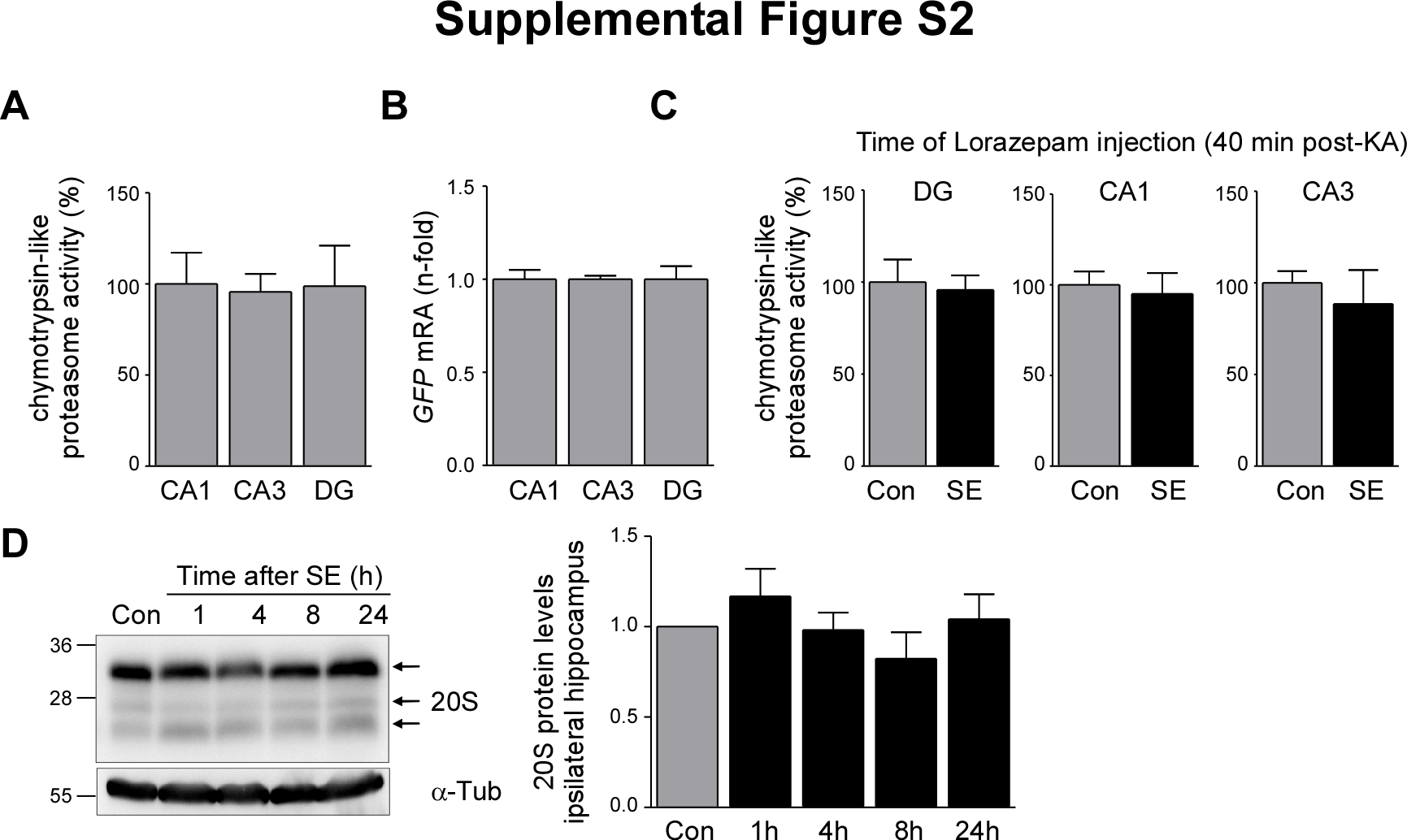


**Fig. S2.** Subfield-specific proteasome activity in the hippocampus **(A**) Graph showing no differences in chymotrypsin-like proteasome activity between the different hippocampal subfields DG, CA3 and CA1 in naive C57/Bl6 wild-type mice (mean ± sem, *p<0.05 by one-way ANOVA with Fischer’s post hoc test; n = 3 per group). (**B)** No difference in *GFP* mRNA levels between different hippocampal subfields (mean ± sem, *p<0.05 by one-way ANOVA with Fischer’s post hoc test; n = 4 per group). (**C)** No significant differences in chymotrypsin-like proteasome activity in the different hippocampal subfields DG, CA1 and CA3 40 min post-KA injection (time-point of lorazepam injection) when compared to vehicle-injected control mice (mean ± sem, *p<0.05 by student’s two-tailed *t*-test; n = 3 per group). (**D)** Representative Western blot (n = 1 per lane) and graph showing similar expression levels of 20S proteasome subunits after *status epilepticus* in the ipsilateral hippocampus. Densitometry was performed on three bands as shown by arrows (mean ± sem, *p<0.05 by one-way ANOVA with Fischer’s post hoc test; n = 4 per group).

**Supplemental Figure 3**

**Decreased polyubiquitination in the hippocampus due to post mortem effect**

To investigate whether post-mortem delays affect polyubiquitination levels, we analysed hippocampal tissue left at room temperature at different time-points post-mortem. Western blotting using the FK-2 antibody showed a dramatic reduction in polyubiquitination shortly after death (**Fig. S3A**). In contrast to what has been observed during status epilepticus, hippocampal levels of the 20S subunit of the proteasome were increased during epilepsy (14 days post-KA injection) (**Fig. S3B**).


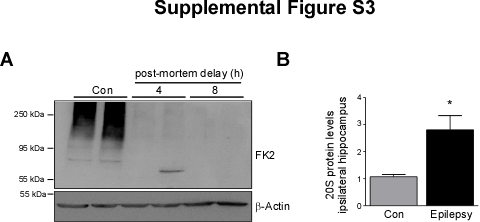


**Fig. S3.** Post-mortem-induced polyubiquitination changes **(A)** Western blot (n = 1 per lane) showing sharp decrease in hippocampal polyubiquitination levels detected with the FK2 antibody after 4 h and 8 h post-mortem interval. (**B**) Graph showing increased expression levels of 20S proteasome subunits during epilepsy in the ipsilateral hippocampus (mean ± sem, *p<0.05 by student’s two-tailed *t*-test; n = 6 (Control) and 8 (epilepsy)).

**Supplemental Figure 4**

**Reduced hippocampal proteasome activity after proteasome inhibitor treatment**

Before commencing our *in vivo* studies using proteasome inhibitors to determine their effects on seizure-induced pathology, we ensured proteasome inhibitors reached the hippocampus in a sufficient dose to reduce proteasome activity. 100 μM and 300 μM epoxomicin delivered via i.c.v. injections in Ub^G76V^-GFP mice increased GFP levels 30 min post-injection in a dose-dependent manner (**Fig. S4A,B**). Furthermore, chymotrypsin-like proteasome activity in the hippocampus was reduced by ~50% 30 min after the i.c.v. injection of 100 μM epoxomicin (**Fig.** **S4C**).


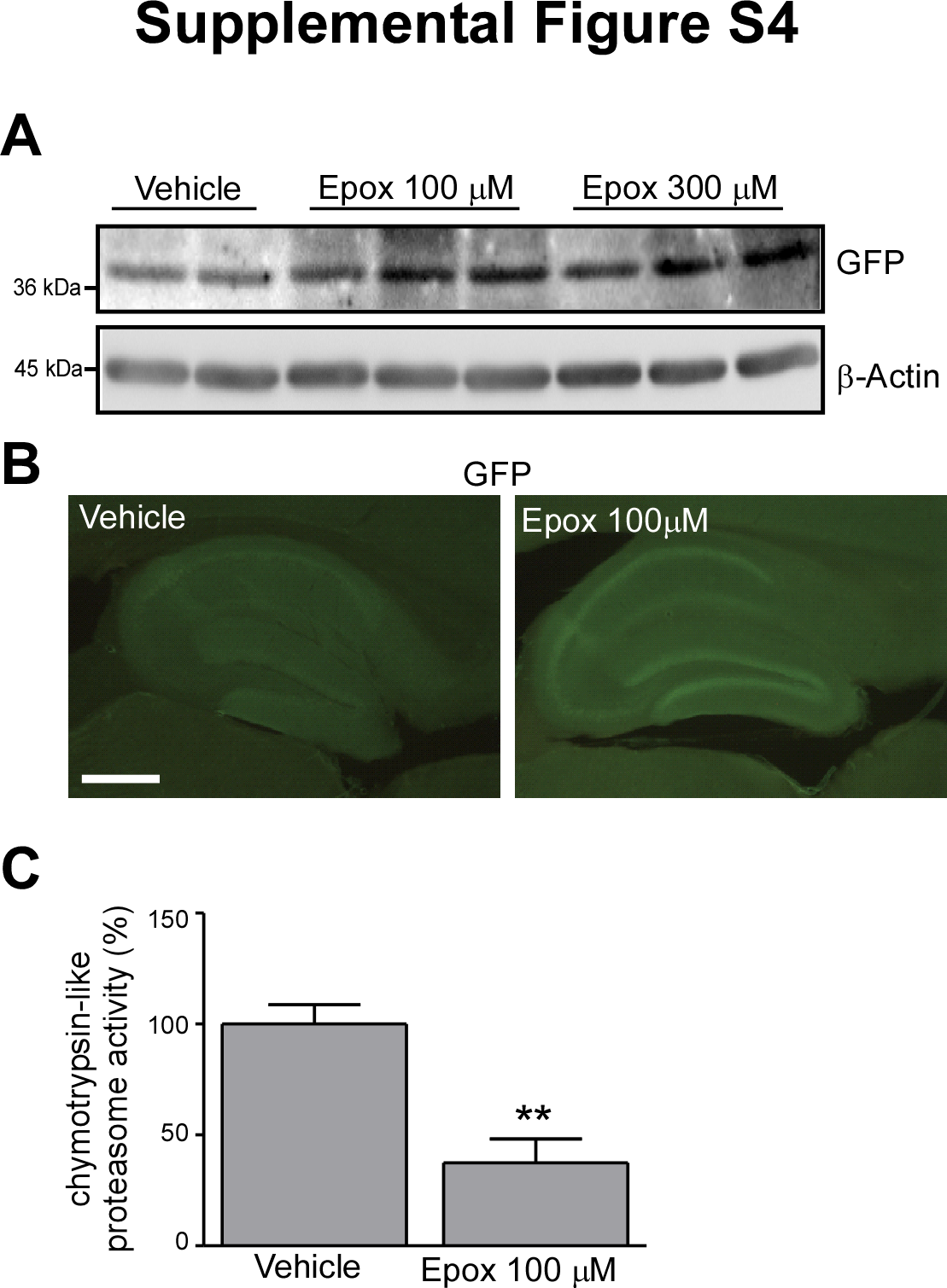


**Fig. S4.** Proteasome inhibitor delivery *in vivo* **(A)** Western blotting (n = 1 per lane) showing increased GFP-protein levels in hippocampal tissue of Ub^G76V^-GFP reporter mice 30 min after i.c.v. injection of different doses of epoxomicin (100 μM and 300 μM). **(B)** Immunofluorescence showing increased hippocampal GFP levels in the hippocampus of transgenic Ub^G76V^-GFP reporter mice 30 min after the injection of 100 μM epoxomicin into the ventricle. **(C)** Graph showing decreased chymotrypsin-like proteasome activity in hippocampal tissue 30 min after i.c.v. injection of 100 μM epoxomicin (mean ± sem, **p<0.001 by student’s two-tailed *t*-test; n = 6 (Vehicle) and 3 (Epox 100 μM)).
